# Housing Improvements and Malaria Risk in Sub-Saharan Africa: A Multi-Country Analysis of Survey Data

**DOI:** 10.1371/journal.pmed.1002234

**Published:** 2017-02-21

**Authors:** Lucy S. Tusting, Christian Bottomley, Harry Gibson, Immo Kleinschmidt, Andrew J. Tatem, Steve W. Lindsay, Peter W. Gething

**Affiliations:** 1 Oxford Big Data Institute, Li Ka Shing Centre for Health Information and Discovery, Nuffield Department of Medicine, University of Oxford, Oxford, United Kingdom; 2 MRC Tropical Epidemiology Group, London School of Hygiene & Tropical Medicine, London, United Kingdom; 3 Department of Infectious Disease Epidemiology, London School of Hygiene & Tropical Medicine, London, United Kingdom; 4 School of Health Sciences, University of Witwatersrand, Johannesburg, South Africa; 5 WorldPop, Department of Geography and Environment, University of Southampton, Southampton, United Kingdom; 6 Flowminder Foundation, Stockholm, Sweden; 7 Department of Biosciences, Durham University, Durham, United Kingdom; Mahidol-Oxford Tropical Medicine Research Unit, THAILAND

## Abstract

**Background:**

Improvements to housing may contribute to malaria control and elimination by reducing house entry by malaria vectors and thus exposure to biting. We tested the hypothesis that the odds of malaria infection are lower in modern, improved housing compared to traditional housing in sub-Saharan Africa (SSA).

**Methods and Findings:**

We analysed 15 Demographic and Health Surveys (DHS) and 14 Malaria Indicator Surveys (MIS) conducted in 21 countries in SSA between 2008 and 2015 that measured malaria infection by microscopy or rapid diagnostic test (RDT). DHS/MIS surveys record whether houses are built with finished materials (e.g., metal) or rudimentary materials (e.g., thatch). This information was used to develop a binary housing quality variable where houses built using finished wall, roof, and floor materials were classified as “modern”, and all other houses were classified as “traditional”. Conditional logistic regression was used to determine the association between housing quality and prevalence of malaria infection in children aged 0–5 y, adjusting for age, gender, insecticide-treated net (ITN) use, indoor residual spraying, household wealth, and geographic cluster. Individual survey odds ratios (ORs) were combined to determine a summary OR using a random effects meta-analysis.

Of 284,532 total children surveyed, 139,318 were tested for malaria infection using microscopy (*n* = 131,652) or RDT (*n* = 138,540). Within individual surveys, malaria prevalence measured by microscopy ranged from 0.4% (Madagascar 2011) to 45.5% (Burkina Faso 2010) among children living in modern houses and from 0.4% (The Gambia 2013) to 70.6% (Burkina Faso 2010) in traditional houses, and malaria prevalence measured by RDT ranged from 0.3% (Senegal 2013–2014) to 61.2% (Burkina Faso 2010) in modern houses and from 1.5% (The Gambia 2013) to 79.8% (Burkina Faso 2010) in traditional houses. Across all surveys, modern housing was associated with a 9% to 14% reduction in the odds of malaria infection (microscopy: adjusted OR 0.91, 95% CI 0.85–0.97, *p* = 0.003; RDT: adjusted OR 0.86, 95% CI 0.80–0.92, *p <* 0.001). This association was consistent regardless of ITN usage. As a comparison, the odds of malaria infection were 15% to 16% lower among ITN users versus non-users (microscopy: adjusted OR 0.84, 95% CI 0.79–0.90, *p <* 0.001; RDT: adjusted OR 0.85, 95% CI 0.80–0.90, *p <* 0.001).

The main limitation of this study is that residual confounding by household wealth of the observed association between housing quality and malaria prevalence is possible, since the wealth index may not have fully captured differences in socioeconomic position; however, the use of multiple national surveys offers the advantage of a large sample size and the elimination of many biases typically associated with pooling observational data.

**Conclusions:**

Housing quality is an important risk factor for malaria infection across the spectrum of malaria endemicity in SSA, with a strength of association between housing quality and malaria similar to that observed between ITN use and malaria. Improved housing should be considered a promising intervention for malaria control and elimination and long-term prevention of reintroduction.

## Abstract 2

Lucy Tusting and colleagues investigate the association between housing quality and malaria infection in children under 5 living in sub-Saharan Africa.

## Author Summary

**Why Was This Study Done?:**

- Insecticide-treated bednets and house spraying have been highly effective in reducing malaria illness and deaths in Africa since 2000, but supplementary approaches are needed for long-term, sustainable intervention.
- Since most malaria mosquitoes bite indoors at nighttime, simple house improvements that prevent mosquitoes from entering homes (e.g., closing eave gaps) can help protect people against malaria infection and disease.
- Although housing improvements show promise as a malaria intervention, few studies with human participants have rigorously evaluated their effect on malaria, nor compared their effectiveness with established malaria interventions.
- We assessed whether modern, improved housing, compared with unimproved housing, was associated with a lower risk of malaria infection in children across Africa.

**What Did the Researchers Do and Find?:**

- We performed a cross-sectional analysis using data from 21 African countries collected in 15 Demographic and Health Surveys and 14 Malaria Indicator Surveys between 2008 and 2015.
- We analysed data from 139,318 total children aged 0–5 y, living in 84,153 households, who were tested for malaria infection using a blood smear or rapid test.
- In individual surveys, the proportion of children with malaria infection in their blood detectable by microscopy ranged from 0.4% to 45.5% among children living in modern houses and from 0.4% to 70.6% among children living in traditional houses.
- Children living in modern, improved housing (e.g., with metal roofs and brick or concrete walls) were less likely (9%–14% lower odds) to be infected with malaria than children living in traditional, unimproved housing, after accounting for differences in levels of urbanisation, wealth, and use of malaria interventions.
- As a comparison, children sleeping under insecticide-treated bednets were less likely (15%–16% lower odds) to be infected with malaria compared to children not sleeping under bednets.

**What Do These Findings Mean?:**

- Improving the quality of housing in Africa may help to reduce the burden of malaria and is a promising intervention for sustainable malaria control.
- Improved housing may have an effect on malaria similar to that of insecticide-treated bednets.
- Community-level experimental studies are needed to confirm our findings in different African settings.

## Introduction

Insecticide-treated nets (ITNs) and indoor residual spraying (IRS) have contributed to a 40% reduction in malaria incidence in endemic Africa since 2000 [1]. These are highly effective methods of vector control, but additional interventions are needed for long-term, sustainable malaria control and elimination. Evidence from some tropical settings indicates that modern, well-built housing protects against malaria [2], with two postulated mechanisms. First, house entry by mosquito vectors is reduced by physical barriers such as closed eaves (the gap between the roof and top of the wall), tiled or metal roofs instead of thatch, and door and window screening [2,3]. Second, daytime indoor temperature is higher in metal-roofed than thatch-roofed houses [4], which may impair parasite development if it exceeds an optimal temperature range [5]. House improvements could be an important pillar of malaria intervention as intersectoral approaches to malaria control and elimination are increasingly encouraged [6,7], especially given the new global target to ensure universal access to adequate, safe, and affordable housing by 2030 [8]. Africa’s rapid economic and population growth—its population is projected to increase from 1.2 billion in 2015 to 2.1 billion in 2050 [9], and it has the world’s fastest rate of urbanisation [10]—presents an unrivalled opportunity to build healthier homes.

Although housing improvements show promise for malaria control, the evidence base remains narrower than that for ITNs and IRS [11]. Observational and phase II experimental studies have examined the household-level association between individual house features and entomological and epidemiological malaria outcomes in a range of African settings [3,12–14], but only one randomised controlled trial has evaluated a housing intervention against epidemiological malaria outcomes [3]. For much of sub-Saharan Africa (SSA), there are no published data, limiting our understanding of how housing may affect malaria transmission across cultures and environments [2], and there has been no comparison between housing and established malaria interventions. Here, we narrow these knowledge gaps through a detailed multi-country analysis of the relationship between housing quality and malaria. Specifically, we use Demographic and Health Surveys (DHS) and Malaria Indicator Survey (MIS) data to test the hypothesis that modern, improved housing is associated with lower odds of malaria infection in children across SSA, compared to traditional, unimproved housing. To our knowledge, this is the most comprehensive study of the relationship between housing quality and malaria across SSA to date, and the first to provide a comparison of housing quality and ITN use in relation to malaria prevalence.

## Methods

### Data Source

The prospective analysis plan is included as S1 Appendix. All analyses were conducted in Stata 13 (StataCorp). We analysed all DHS and MIS surveys conducted in SSA and published up to 1 July 2016 that included data collection on malaria infection in children, measured by rapid diagnostic test (RDT) or microscopy using thick or thin blood smears. DHS and MIS surveys are cross-sectional surveys designed to collect nationally representative health and sociodemographic data [15,16]. Each survey is administered approximately every 5 y in each target country, meaning that multiple independent surveys conducted in different years may be available for individual countries. The survey designs are based on a set of standard questionnaires that are adapted by country, so that the variables collected vary between each survey. The surveys have a stratified two-stage cluster design in which (i) primary sampling units (PSUs) are randomly selected from census data and (ii) households are randomly selected within PSUs from an updated enumeration list.

### Housing Quality

DHS and MIS surveys classify wall, roof, and floor materials as “natural”, “rudimentary”, or “finished” [17]. While definitions vary by country and year, finished wall materials typically include cement, stone with lime or cement, bricks, cement blocks, covered adobe, and wood planks or shingles, and natural or rudimentary wall materials typically include cane, palm, dirt, bamboo or stone with mud, uncovered adobe, plywood, cardboard, and reused wood planks. Finished roof materials typically include metal, wood, calamine or cement fibre, ceramic tiles, cement, and roofing shingles, and natural or rudimentary roof materials typically include thatch, palm leaf, sod, rustic matting, bamboo, wood planks, and cardboard. Finished floor materials include parquet or polished wood, vinyl or asphalt strips, ceramic tiles, cement, and carpet, and natural or rudimentary floor materials include earth, sand, dung, wood planks, palm, and bamboo [17]. We used the DHS/MIS definitions of finished, rudimentary, and natural materials to create a binary housing quality variable comparing “modern” with “traditional” housing. Houses built with a finished wall, finished roof, and finished floor material were classified as modern, and all other houses were classified as traditional. However, in contrast to the DHS and MIS methods, we considered wood and wood shingles to be natural materials (not finished) because they may contain openings permeable to mosquitoes. We also considered sheet metal always to be a finished material because it is likely to increase indoor temperature and reduce house entry points for mosquitoes (S2 Appendix) [18]. We used the chi-squared test to compare the prevalence of modern housing in urban and rural areas and among wealthier and poorer households.

### Insecticide-Treated Net Use

ITNs were defined as (i) long-lasting insecticidal nets that were ≤3 y old at the time of survey or (ii) conventional ITNs that were ≤1 y old or were retreated within the year before the survey [19].

### Household Wealth

DHS and MIS household wealth index scores are developed using principal component analysis that typically includes variables describing durable asset ownership, access to utilities and infrastructure, and house construction materials [20]. We constructed a modified wealth index for each survey using the variables described in S2 Appendix. This index was to adjust for confounding and as such did not include variables related to house construction, to avoid inducing a circularity that could cause an underestimation of the association between housing quality and malaria. The assets included in the index varied by survey because of differences in survey design and because we excluded assets where <5% or >95% of households owned the asset [21]. As well as being assigned a wealth index score, households were ranked by their wealth score and grouped into tertiles to give a categorical measure of socioeconomic position for each survey.

### Association between Housing Quality and Malaria Infection

For each national DHS or MIS survey, we modelled the association between housing quality (modern versus traditional) and the odds of malaria infection in children aged 0–5 y using conditional logistic regression, adjusting for (i) age, (ii) gender, (iii) ITN use, (iv) IRS in the past 12 mo, and (v) household wealth index score. The analysis was restricted to the age group 0–5 y because DHS/MIS surveys measure malaria parasitaemia within this child age range only. Age and household wealth index score were included as restricted cubic spline functions with six knots (i.e., six cut points) located at percentiles 5, 23, 41, 59, and 95 [22]. The restricted cubic spline splits the continuous variables into categories and uses separate cubic relationships to model the association within each category, except in the first and last category, where a linear relationship is assumed. The restricted cubic spline allows non-linearities to be modelled while keeping variables continuous. We used conditional logistic regression to enable the association between house type and malaria infection to be estimated within geographical clusters (PSUs), so that the analysis eliminated confounding due to inter-cluster variation in malaria transmission intensity and house construction (e.g., traditional houses might be more common in clusters with high transmission). Individual survey odds ratios (ORs) were combined to determine a summary OR across all surveys using a random effects meta-analysis. Individual and summary ORs are displayed in forest plots. We used random effects meta-regression to compare ORs between children who used ITNs and those who did not, i.e., to test for effect modification by ITN use. In this analysis, ORs were adjusted for (i) age, (ii) gender, (iii) IRS in the past 12 mo, (iv) household wealth index score, and (v) geographic cluster.

### Association between Insecticide-Treated Net Use and Malaria Infection

To compare the observed association between housing quality and malaria with that between ITN use and malaria, we additionally modelled the association between reported ITN use the night before the survey and the odds of malaria infection, adjusting for (i) age, (ii) gender, (iii) IRS in the past 12 mo, (iv) household wealth index score, and (v) house type. Age and household wealth index score were included as restricted cubic spline functions with six knots. We used a conditional logistic model to adjust for cluster-level confounding.

## Results

### Study Population

A total of 15 DHS surveys and 14 MIS surveys conducted between 2008 and 2015 were included in the analysis (Table 1). These surveys were conducted in 21 countries: Angola, Benin, Burkina Faso, Burundi, Cameroon, Côte d’Ivoire, Democratic Republic of the Congo, The Gambia, Ghana, Guinea, Kenya, Liberia, Madagascar, Malawi, Mali, Mozambique, Nigeria, Rwanda, Senegal, Togo, and Uganda. A further two surveys (Angola 2006–2007 MIS and Rwanda 2007–2008 DHS) that measured malaria parasitaemia were excluded from the analysis because data on main wall and roof material were not recorded. Of 245,806 total households surveyed, 149,803 households had at least one resident child aged 0–5 y, yielding a total population of 284,532 children aged 0–5 y. Of these, 139,318 total children resident in 84,153 households were tested for malaria infection using microscopy (*n* = 131,652) or RDT (*n* = 138,540). Across all surveys, the average age of the children was 2.5 y, and 49.5% were female.

**Table 1:** Characteristics of surveys included in the analysis.

Survey	Survey Type	Household-Level Characteristics^a^				Child-Level Characteristics^b^					
		*N*	Modern House (Percent)	Urban Residence (Percent)	IRS in Past 12 mo (Percent)	*N*	Mean Age (Years)	Female (Percent)	Slept under ITN Previous Night (Percent)	Parasite Rate (Percent) (Total Slides or RDTs)	
										Microscopy	RDT
Angola 2011^†^	MIS	8,391	34.0	39.5	—	9,681	2.4	50.1	24.5	9.8 (3,431)	12.6 (3,432)
Benin 2011–2012	DHS	17,422	40.7	40.8	7.5	17,489	2.6	49.0	69.8	30.5 (4,638)	27.6 (4,695)
Burkina Faso 2010	DHS	14,424	28.4	30.6	1.5	16,969	2.4	49.2	48.5	65.0 (6,102)	75.6 (6,125)
Burkina Faso 2014	MIS	6,448	22.2	20.4	0.8	8,419	2.5	49.1	75.2	47.7 (6,117)	64.4 (6,154)
Burundi 2012	MIS	4,866	15.0	18.1	4.8	4,985	2.4	50.3	53.2	16.2 (3,722)	20.6 (3,750)
Cameroon 2011	DHS	14,214	40.3	47.2	3.2	14,276	2.4	50.3	13.2	—	33.5 (6,605)
Côte d’Ivoire 2011–2012	DHS	9,686	60.4	41.4	1.4	9,742	2.5	50.0	37.4	17.7 (4,044)	46.6 (4,215)
DRC 2013–2014^†^	DHS	18,171	12.5	30.0	—	22,059	2.4	50.4	52.3	26.3 (8,186)	36.0 (8,219)
The Gambia 2013	DHS	6,217	64.0	49.8	42.0	10,701	2.4	49.1	45.3	0.5 (3,481)	1.8 (3,298)
Ghana 2014	DHS	11,835	66.0	50.2	18.4	7,341	2.4	48.1	47.4	30.7 (3,197)	42.2 (3,191)
Guinea 2012	DHS	7,109	46.9	35.2	1.7	8,531	2.5	48.6	27.1	43.4 (3,220)	45.6 (3,215)
Kenya 2015^†^	MIS	6,481	43.3	46.1	—	4,724	2.6	49.7	56.1	5.9 (4,105)	10.0 (4,095)
Liberia 2009^†^	MIS	4,162	28.4	45.3	—	5,557	2.5	49.9	28.8	33.0 (4,968)	37.1 (4,960)
Liberia 2011	MIS	4,162	34.1	46.0	10.7	4,340	2.5	49.8	36.5	32.0 (3,081)	51.3 (3,187)
Madagascar 2011	MIS	8,094	18.5	25.6	46.1	8,109	2.5	49.1	74.7	4.3 (6,836)	6.4 (6,874)
Madagascar 2013	MIS	8,574	13.1	25.7	35.5	7,306	2.5	49.0	53.8	7.3 (6,151)	8.2 (6,232)
Malawi 2012	MIS	3,404	30.6	31.0	8.6	2,813	2.4	52.5	57.2	24.8 (2,112)	38.4 (2,115)
Malawi 2014	MIS	3,405	35.0	35.6	6.0	2,621	2.4	49.9	68.9	26.6 (1,928)	30.1 (1,921)
Mali 2012–2013	DHS	10,107	18.8	27.4	8.4	12,882	2.5	49.1	68.2	50.2 (5,646)	45.0 (5,706)
Mozambique 2011	DHS	13,919	23.3	36.6	21.3	12,683	2.4	50.1	33.1	29.9 (4,898)	33.9 (4,916)
Nigeria 2010	MIS	5,895	49.0	33.0	1.0	6,941	2.4	49.1	29.8	38.1 (5,137)	47.2 (5,147)
Rwanda 2010^†^	DHS	12,540	15.4	16.0	—	10,697	2.6	49.2	67.8	1.5 (4,950)	2.6 (4,893)
Senegal 2008–2009^†^	MIS	10,651	35.1	31.0	—	23,105	2.5	48.9	32.1	6.7 (4,138)	12.0 (4,032)
Senegal 2010–2011	DHS	7,904	43.9	37.5	12.4	15,752	2.5	48.6	44.0	4.0 (4,698)	3.4 (4,716)
Senegal 2012–2013	DHS	4,177	49.5	39.2	15.5	8,746	2.5	50.0	48.6	4.2 (7,266)	4.7 (7,316)
Senegal 2013–2014	DHS	4,233	48.3	39.1	11.2	8,432	2.4	49.8	49.2	1.9 (6,762)	1.8 (6,762)
Togo 2013–2014^†^	DHS	9,549	51.2	38.1	—	8,583	2.5	49.6	43.5	39.5 (3,888)	41.2 (3,868)
Uganda 2009^†^	MIS	4,421	23.5	14.9	—	4,940	2.5	49.9	31.9	43.6 (4,011)	53.1 (3,998)
Uganda 2014–2015	MIS	5,345	25.2	20.9	8.1	6,108	2.5	51.0	73.6	19.7 (4,939)	32.8 (4,903)

^a^All surveyed households with at least one household member.

^b^All surveyed children aged 0–5 y.

^†^Survey did not collect data on IRS in the past 12 mo.

DHS, Demographic and Health Surveys; DRC, Democratic Republic of the Congo; IRS, indoor residual spraying; ITN, insecticide-treated net; MIS, Malaria Indicator Survey; RDT, rapid diagnostic test.

### Housing Quality and Intervention Coverage

The proportion of houses classified as modern (versus traditional) was 34% overall and ranged from 12% (Democratic Republic of the Congo 2013–2014) to 66% (Ghana 2014). The proportion of urban (versus rural) households was 35% overall and ranged from 15% (Uganda 2009) to 50% (Ghana 2014) (Table 1). Across all surveys, a greater proportion of houses were classified as modern in urban areas (66%) than in rural areas (17%) (*p <* 0.001) and in the wealthiest tertile of households (68%) than in the middle (26%) and poorest tertiles (9%) (*p <* 0.001). ITN usage (the proportion of children reported to have slept under an ITN the night before the survey) was 47% overall and ranged from 13% (Cameroon 2011) to 75% (Burkina Faso 2014) (Table 1). There was no difference in the proportion of children sleeping under ITNs in urban areas compared to rural areas. IRS coverage (the proportion of households with IRS in the past 12 mo) was 13% overall and ranged from less than 1% (Burkina Faso 2014) to 46% (Madagascar 2011). IRS data were not recorded in eight of 29 surveys.

### Malaria Infection

A total of 131,652 blood smears were taken in 28 surveys, of which 30,625 (23%) had visible malaria parasites (Table 1). Malaria infection prevalence measured by microscopy ranged from 0.5% (The Gambia 2013) to 65% (Burkina Faso 2010). A total of 138,540 RDTs were done in 29 surveys, of which 40,541 (29%) were positive. Infection prevalence by RDT ranged from 2% (The Gambia 2013; Senegal 2013–2014) to 76% (Burkina Faso 2010).

### Risk Factors for Malaria Infection

#### Housing quality

**Fig 1:**
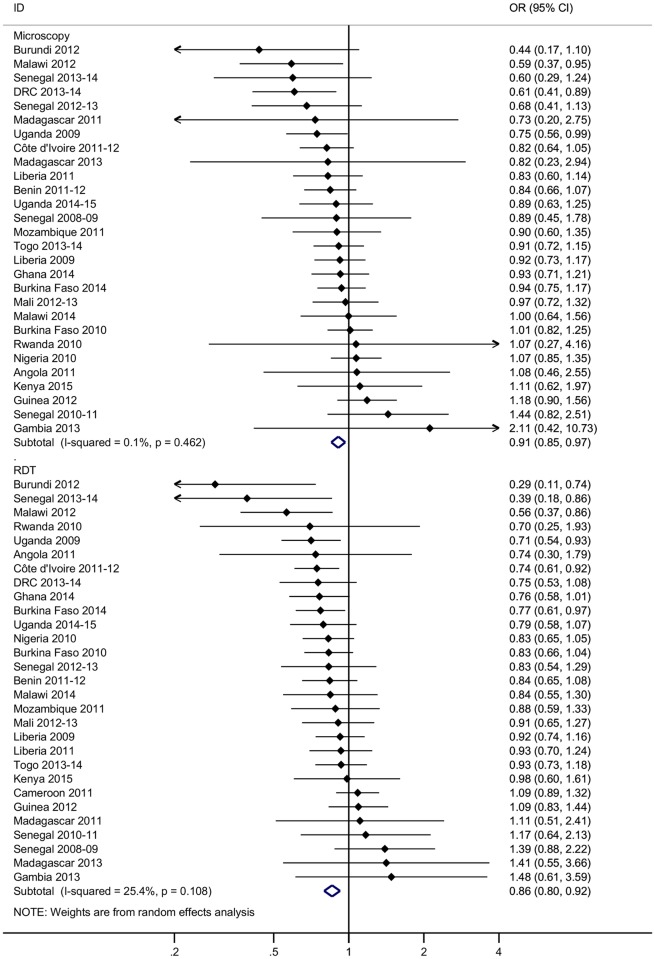
Reduction in the odds of malaria infection in children aged 0–5 y living in modern houses in sub-Saharan Africa. Values to the left of the vertical line representing the null value indicate a reduction in the odds of malaria infection in modern housing compared to traditional housing. Data are taken from 15 Demographic and Health Surveys and 14 Malaria Indicator Surveys conducted between 2008 and 2015. Houses built with a finished wall, finished roof, and finished floor material were classified as modern, and all other houses were classified as traditional (S2 Appendix). ORs are adjusted for age, gender, insecticide-treated net use, indoor residual spraying in the past 12 mo (where measured), household wealth, and geographic cluster. Summary effects are from random effects analysis. Sub-groups show diagnostic test. Error bars show 95% confidence intervals. DRC, Democratic Republic of the Congo; OR, odds ratio; RDT, rapid diagnostic test.

Across all surveys, adjusting for age, gender, ITN use, IRS coverage (where measured), household wealth, and cluster-level variables such as urban/rural status, modern housing was associated with a 9% reduction in the odds of malaria infection as measured by microscopy (adjusted OR 0.91, 95% CI 0.85–0.97, *p* = 0.003) and a 14% reduction in the odds of malaria infection as measured by RDT (adjusted OR 0.86, 95% CI 0.80–0.92, *p <* 0.001) (Fig 1). Unadjusted ORs and malaria prevalence in exposed and unexposed groups are provided by survey in S3 Appendix. Malaria prevalence measured by microscopy ranged from 0.4% (Madagascar 2011) to 45.5% (Burkina Faso 2010) among children living in modern houses and from 0.4% (The Gambia 2013) to 70.6% (Burkina Faso 2010) among those living in traditional houses; malaria prevalence measured by RDT ranged from 0.3% (Senegal 2013–2014) to 61.2% (Burkina Faso 2010) in modern houses and from 1.5% (The Gambia 2013) to 79.8% (Burkina Faso 2010) in traditional houses. Although modern housing was associated with a greater reduction in the odds of malaria infection among children using ITNs (microscopy: adjusted OR 0.87, 95% CI 0.79–0.96, *p* = 0.007; RDT: adjusted OR 0.83, 95% CI 0.75–0.92, *p <* 0.001) than among children not using ITNs (microscopy: adjusted OR 0.92, 95% CI 0.82–1.03, *p* = 0.14; RDT: adjusted OR 0.91, 95% CI 0.82–1.01, *p* = 0.07), there was no evidence of effect modification by ITN use (S4 Appendix).

#### Insecticide-treated net use

**Fig 2:**
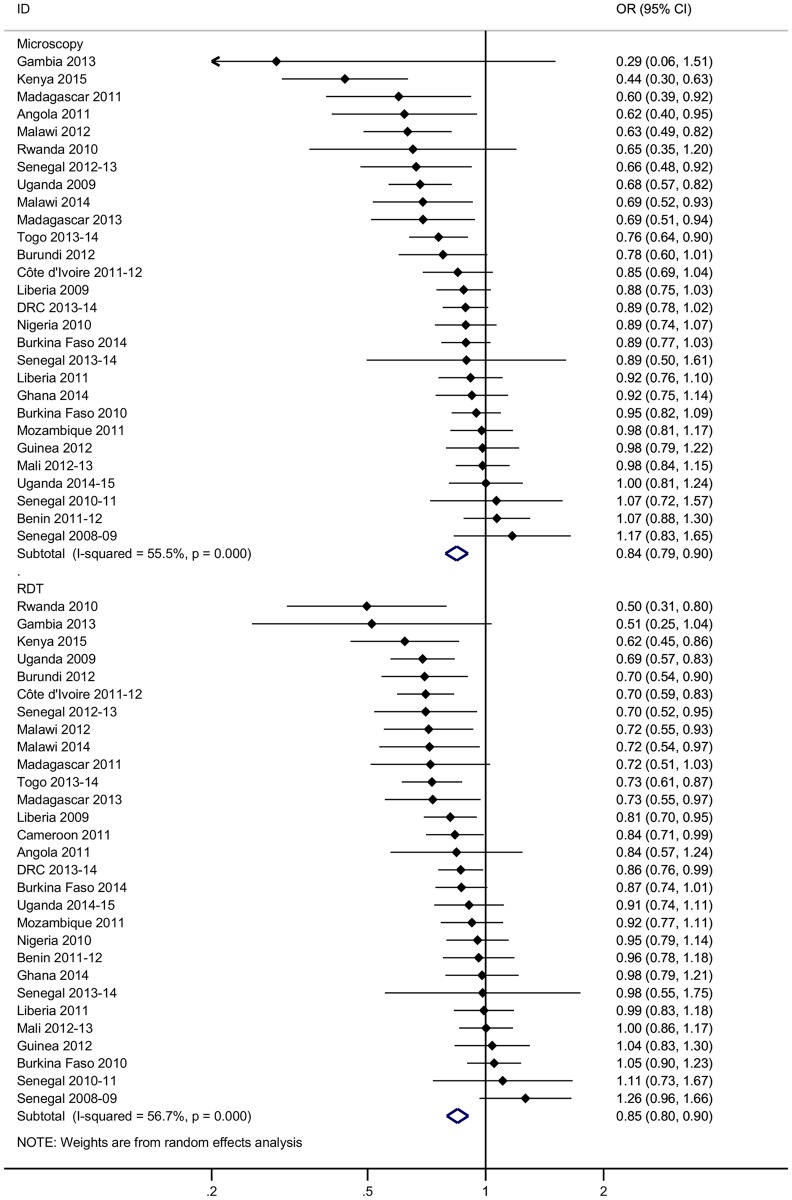
Reduction in the odds of malaria infection in children aged 0–5 y sleeping under insecticide-treated nets in sub-Saharan Africa. Values to the left of the vertical line representing the null value indicate a reduction in the odds of malaria infection in users of insecticide-treated nets compared to non-users. Data are taken from 15 Demographic and Health Surveys and 14 Malaria Indicator Surveys conducted between 2008 and 2015. ORs are adjusted for age, gender, indoor residual spraying in the past 12 mo (where measured), household wealth, house type, and geographic cluster. Summary effects are from random effects analysis. Sub-groups show diagnostic test. Error bars show 95% confidence intervals. DRC, Democratic Republic of the Congo; OR, odds ratio; RDT, rapid diagnostic test.

Children who were reported to have slept under an ITN the previous night had 15% to 16% lower odds of malaria infection than those not reported to have done so, adjusting for age, gender, IRS coverage (where measured), household wealth, house type, and geographic cluster (microscopy: adjusted OR 0.84, 95% CI 0.79–0.90, *p <* 0.001; RDT: adjusted OR 0.85, 95% CI 0.80–0.90, *p <* 0.001) (Fig 2).

## Discussion

We have conducted a comprehensive analysis of the relationship between housing quality and malaria across SSA and, to our knowledge, provided the first direct comparison with ITNs. Our analysis of 29 DHS and MIS surveys found living in a modern house to be associated with 9% to 14% lower odds of malaria infection in children aged 0–5 y, compared to living in a traditional house. As a comparison, ITN use was associated with a 15% to 16% reduction in the odds of malaria infection. Our study indicates that poor housing quality is an important risk factor for malaria in SSA and that improved housing is a promising intervention for malaria control and elimination and prevention of reintroduction.

Well-built housing with fewer entry points for mosquitoes can help reduce malaria transmission by lowering human exposure to infectious bites [3,23]; specific features thought to deter *Anopheles gambiae* entry include closed eaves [12], the presence of a ceiling, and screened doors and windows [3,24]. In addition, daytime indoor temperature may be higher in houses with metal roofs and no ceilings than in traditional thatched homes [4], which, if exceeding a certain threshold [5], could lower mosquito survival [5] and reduce parasite development within the mosquito [5,25]. Our analysis was limited to assessing wall, roof, and floor material only, but finished materials are often associated with modern housing styles that incorporate other protective features such as closed eaves [14]. Since we examined only those differences in housing quality naturally present within communities, a larger difference in infection prevalence may be observed with direct interventions that specifically target house entry by mosquitoes. The results of this study concur with those of a recent systematic review and meta-analysis of housing quality and malaria, which found that residents of modern houses had 47% lower odds of malaria infection and 45%–65% lower odds of clinical malaria compared to residents of traditional houses [2]. While the reduction in the odds of malaria infection associated with modern housing was greater among ITN users than non-users, the meta-regression gave no evidence that the effect of house type varied by ITN use. Further research is needed to understand the interaction between house design and other indoor interventions. Overall, our findings are consistent with incremental house improvements having a major cumulative potential to reduce malaria transmission at the continental scale, as SSA continues to develop economically.

This is the first study to our knowledge to provide a comparison between housing and ITNs across a range of transmission settings in SSA, and the findings are likely to be generalisable across the continent. We found ITN use to be associated with a 15% to 16% reduction in the odds of malaria infection compared to non-use. Our analysis builds on an earlier study of the association between ITN use and malaria prevalence in children aged 0–5 y, which analysed seven DHS and MIS surveys and found that sleeping under an ITN was associated with a 24% reduction in malaria infection prevalence (adjusted OR 0.76, 95% CI 0.58–0.99, *p* = 0.001) [26]. We included 22 more surveys—and consequently a much larger sample of children—and may have better controlled for confounding due to geographical differences in transmission intensity, survey timing, and urbanicity by adjusting for geographical cluster, while the previous study adjusted for those factors individually. The association between ITNs and malaria prevalence we observed is similar to the effect found in a Cochrane review of randomized trials, where ITNs reduced malaria prevalence by 13% [27]. The association between ITNs and malaria prevalence provides a useful baseline against which housing quality compares favourably.

In response to the Sustainable Development Goals, it is now widely acknowledged that malaria must be tackled across sectors, in conjunction with national plans for economic development [6,7] and the control of other vector-borne diseases [28]. If effective in reducing malaria morbidity, improved housing could reduce transmission by vectors that have developed resistance to the insecticides currently available for IRS and long-lasting insecticidal nets [29] and therefore provide a sustainable means to contribute to malaria elimination. Rapid population growth, urbanisation, and economic development in SSA also present a major opportunity to reduce malaria transmission [30]. The urban population of Africa is expected to increase from 400 million in 2010 to 1.26 billion by 2050 and reach 50% of the total population by 2035 [31]. This growth, in the context of the New Urban Agenda for sustainable global urban development [10] and the wider suppression of malaria transmission in urban environments [32], provides an opportunity for effective long-term intervention against malaria. Indeed, we observed no difference in ITN use between urban and rural areas, but found modern housing to be more prevalent in urban areas than rural areas, and among wealthier households than poorer households. This suggests that improved housing as a malaria intervention may be complementary to the processes of urbanisation and economic development ongoing in SSA. However, this observation should not be universally generalised since urban slums can contain dense housing of poor quality, with poor drainage and environmental management, enabling malaria vectors to proliferate. Reducing urban poverty and upgrading slums is therefore of central importance to reducing malaria and other environment-linked health outcomes. In addition, metal-roofed housing is not a traditional African architectural style and may be hotter in the daytime than existing housing, so there is an ongoing need to work with housing programmes and urban planners to incorporate protective features into locally appropriate and comfortable housing designs, so that the intervention may be universally acceptable.

This study has several limitations. First, although the analysis controlled for household wealth, there may still be residual confounding if the wealth index did not fully capture differences in socioeconomic position that are associated with housing quality. While it is possible that the higher indoor temperature in metal-roofed housing without ceilings reduces mosquito survival, it is also feasible that wealthier households are more likely to contain fans or air conditioning that reduce indoor feeding by vectors. Reverse causation is also possible, if households with a greater malaria burden are consequently poorer and less able to invest in improving their homes. Second, DHS and MIS surveys are designed to be nationally representative, but matching on PSU caused observations to be dropped when the outcome did not vary within sampling units, reducing the total sample size. Therefore, our study may underestimate differences between residents of modern and traditional homes, and ITN users and non-users. Third, we observed that modern housing was associated with increased odds of malaria infection in some surveys and found an inconsistent pattern in the association of house type with malaria prevalence over time within some countries (e.g., Senegal). It is possible that our definition of the housing quality variable was sensitive to small differences in questionnaire design and did not fully capture those differences in housing quality relevant to malaria. Differences within country surveys may also have resulted from sampling differences. Together, these observations highlight the need for field studies to clarify the potential protective effect of improved housing in specific settings. Yet, overall, the use of national survey data offers the advantage of eliminating many biases typically associated with pooling observational data, including publication, selection, and measurement bias and selective outcome reporting, which were perceived to be problematic in a recent systematic review and meta-analysis of housing and malaria [2].

In conclusion, housing quality is an important risk factor for malaria across a range of transmission settings in SSA. Incremental improvements to African housing linked to socioeconomic development should be considered a major opportunity to reduce malaria transmission and a promising strategy to contribute to achieving and sustaining elimination in the long term.

## Supporting Information

S1 Appendix Prospective analysis plan. (PDF)

S2 Appendix Classification of house type and development of the modified wealth index. (PDF)

S3 Appendix Association between house type, insecticide-treated net use, and malaria infection in children aged 0–5 y in sub-Saharan Africa (unadjusted analyses). (PDF)

S4 Appendix Association between house type and malaria infection in children aged 0–5 y in sub-Saharan Africa, stratified by insecticide-treated net use. (PDF)

S5 Appendix STROBE Statement. (PDF)

## Abbreviations

DHS: Demographic and Health Surveys
IRS: indoor residual spraying
ITN: insecticide-treated net
MIS: Malaria Indicator Survey
OR: odds ratio
PSU: primary sampling unit
RDT: rapid diagnostic test
SSA: sub-Saharan Africa
