# Supplementary material for: Housing Improvements and Malaria Risk in Sub-Saharan Africa: A Multi-Country Analysis of Survey Data
Source: PLoS Med. 2017 Feb 21;14(2):e1002234. doi: 10.1371/journal.pmed.1002234 (PMC5319641; doi:10.1371/journal.pmed.1002234)
Supplement: S1 Appendix — (PDF) [file pmed.1002234.s001.pdf]

S1 Appendix: Prospective Analysis Plan (27th November 2015)

The association between house quality and malaria in sub-Saharan Africa: a multi-country analysis

Background

While long-lasting insecticide-treated nets (LLINs) and indoor residual spraying (IRS) have contributed to a 30% decline in malaria incidence in sub-Saharan Africa (SSA) since 2000,[1] house improvements show considerable potential to support sustainable intervention.[3] Though long neglected in malaria control, recent studies indicate that modern, well-built housing can protect residents in many tropical countries by reducing house entry by mosquito vectors.[4,5] Beneficial features include closed eaves (the gap between the top of the wall and the roof), tiled or metal roofs instead of thatch, and door and window screening.[5] [6] Furthermore, rapid economic growth in SSA today presents a unique opportunity to improve the built environment to reduce malaria transmission.;[7,8] the population of SSA is projected to double between 2010 and 2040 to nearly two billion, with 144 million new houses needed by 2030 in rural areas alone.[9] Yet despite the potential contribution of house improvements to malaria control, we lack data to guide research and policy decisions.[10] Studies have examined the household-level association between specific house features and entomological and epidemiological malaria outcomes in individual African settings.[4,6,11,12] However, to date only one published randomised control trial has evaluated a housing intervention against malaria.[6] Indeed, a recent systematic review of housing and malaria highlighted the paucity of well-conducted studies, the high risk of bias within and across studies and the lack of data from many geographical regions.[5]

In this study we will conduct the first multi-country analysis of the relationship between house quality and malaria. Specifically, we will exploit routinely collected household surveys to test the hypothesis that modern housing is associated with a lower odds of malaria infection in children across SSA, compared to traditional housing. In doing so, this study will address a critical need to evaluate the association between housing and malaria across SSA and to elucidate how this relationship varies across local building styles and materials, urbanicity, transmission intensity and intervention coverage.

Analysis plan

Data source: We will extract data from the Demographic and Health Surveys (DHS) and Malaria Indicator Surveys (MIS).[14, 15] The DHS and MIS typically collect health and sociodemographic data every five years and select households using a two-stage random cluster sampling strategy. We will include in our analysis all DHS and MIS conducted in SSA that collected data on malaria infection in children, measured by rapid diagnostic test (RDT) or microscopy, as well as house construction materials and all covariates included in the analyses.

Malaria infection: Malaria infection in children aged ≤5 years was determined in surveys using a rapid diagnostic test (RDT) and/or microscopy using thick or thin blood smears.

Housing quality: Houses will be classified as traditional (built of rudimentary materials) or modern (built of non-rudimentary materials). In SSA we will consider rudimentary building materials to be earth, sand or dung floors; cane, palm leaves or sod roofs; and cane, palm, trunks, brush or mud walls, except in areas of exceptionally high rainfall such as Equatorial Guinea, where concrete may instead be a rudimentary wall material.[13,14] We will use floor material alone to classify houses where surveys do not include roof and wall material.

ITN use: We will consider ITNs to be (i) LLINs that were ≤3 years old at the time of survey or (ii) conventional ITNs that were ≤1 year old or were retreated within the year before the survey.

Household wealth: DHS/MIS survey datasets divide households into wealth quintiles using wealth index scores that are calculated by entering a range of variables into a factor analysis. These variables vary between surveys but typically include durable asset ownership, access to utilities and infrastructure (e.g. sanitation facilities) and housing characteristics. However, the inclusion of housing characteristics in the wealth index may underestimate the association between housing quality and malaria infection, when controlling for household wealth. For each country, we will therefore replicate standard DHS methods to re-construct country wealth indices,[14] excluding housing characteristics. We will also create wealth tertiles instead of quintiles to reduce the number of parameters to be modelled.

Association between housing quality and malaria infection: We will follow a similar approach to a previous evaluation of LLINs to evaluate the association between house type and malaria.[15] For each survey, we will model the association between house type and the odds of malaria infection in children aged <5 years using conditional logistic regression, adjusting for (i) child's age (<2 years, 2 to <5 years), (ii) LLIN use, (iii) IRS in the past 12 months and (iv) household wealth. The conditional logistic model will match on PSU and hence the analysis will be "within PSU" to control for variation in transmission intensity, urban/rural status and survey timing between PSUs.

Individual survey odds ratios will be combined to determine a pooled odds ratio (OR) across all surveys using random-effects. Survey and pooled ORs will be displayed in a forest plot. We will use meta-regression to assess effect modification by LLIN use and IRS coverage.

References

1. Bhatt S, Weiss D, Cameron E, et al. The effect of malaria control on Plasmodium falciparum in Africa between 2000 and 2015. *Nature* 2015; **526**: 207-11.
2. Hemingway J. The role of vector control in stopping the transmission of malaria: threats and opportunities. *Phil Trans R Soc B* 2014; **369**: 20130431.
3. RBM. Consensus statement on housing and malaria. Geneva: Roll Back Malaria Housing and Malaria Work Stream, 2015.
4. Wanzirah H, Tusting LS, Arinaitwe E, et al. Mind the gap: house construction and the risk of malaria in Ugandan children. *PLOS ONE* 2015; **10**: e0117396.
5. Tusting LS, Ippolito M, Kleinschmidt I, et al. The evidence for improving housing to reduce malaria: a systematic review and meta-analysis. *Malaria J* 2015; **14**: 209.
6. Kirby M, Ameh D, Bottomley C, et al. Effect of two different house screening interventions on exposure to malaria vectors and on anaemia in children in The Gambia: a randomised controlled trial. *Lancet* 2009; **374**: 998-1009.
7. Tusting LS, Willey B, Lucas H, et al. Socioeconomic development as an intervention against malaria: a systematic review and meta-analysis. *Lancet* 2013; **382**: 834.
8. Tatem A, Gething P, Smith D, Hay S. Urbanization and the global malaria recession. *Malaria J* 2013; **12**: 133.
9. UN-Habitat. State of African Cities 2014: Re-imagining sustainable urban transitions. Nairobi: UN Habitat, 2014.
10. Anderson L, Simpson D, Stephens M. Durable Housing Improvements to Fight Malaria Transmission: Can we learn new strategies from past experience? . Atlanta: Habitat for Humanity International – Global Programs Department, 2014.
11. Njie M, Dilger E, Lindsay SW, Kirby MJ. Importance of eaves to house entry by Anopheline, but not Culicine, mosquitoes. *Journal of Medical Entomology* 2009 **46**: 977-84.
12. Liu JX, Bousema T, Zelman B, et al. Is housing quality associated with malaria incidence among young children and mosquito vector numbers? Evidence from Korogwe, Tanzania *PLoS ONE* 2014; DOI: 10.1371/journal.pone.0087358.
13. Bradley J, Rehman AM, Schwabe C, et al. Reduced prevalence of malaria infection in children living in houses with window screening or closed eaves on Bioko Island, Equatorial Guinea. *PLoS ONE* 2013; **8**: e80626.
14. Rutstein SO. Steps to constructing the new DHS Wealth Index. Rockville, MD: ICF International; 2015.
15. Lim SS, Fullman N, Stokes A, et al. Net Benefits: A multicountry analysis of observational data examining associations between insecticide-treated mosquito nets and health outcomes. *PLoS Med* 2011; **8**: e1001091.
