# Supplementary material for: Housing Improvements and Malaria Risk in Sub-Saharan Africa: A Multi-Country Analysis of Survey Data
Source: PLoS Med. 2017 Feb 21;14(2):e1002234. doi: 10.1371/journal.pmed.1002234 (PMC5319641; doi:10.1371/journal.pmed.1002234)
Supplement: S2 Appendix — (PDF) [file pmed.1002234.s002.pdf]

S2 Appendix. Classification of house type and development of the modified wealth index

Country	Survey type	Roof		Walls		Floor		Wealth index variables
		Rudimentary	Non-rudimentary	Rudimentary	Non-rudimentary	Rudimentary	Non-rudimentary	
Angola 2011	MIS	Palm; bamboo; mat; wood planks; tarpaulin; plastic; other	Zinc; metal; asbestos; ceramic tiles; concrete; cement	Straw; mats; cardboard; plastic; sticks and mud; clay blocks; cane; palm; trunks; used wood; wood planks; other	Concrete; stone blocks; bricks	Clay; sand; dung; tablets; wood planks; other	Parquet; polished wood; ceramic; mosaic; tiles; cement; carpet	(1) source of drinking water; (2) toilet facility; (3) cooking fuel; (4) electricity; ownership of a: (5) radio, (6) television, (7) refrigerator, (8) bicycle, (9) scooter, (10) car; (11) mobile telephone; (12) watch
Benin 2011-12	DHS	No roof; thatch; palm leaf; sod; rustic mat; palm; bamboo; wood planks; cardboard; wood; roofing shingles; other	Metal; calamine or cement fibre; ceramic tiles; cement	No walls; cane; palm; trunks; dirt; bamboo with mud; stone with mud; uncovered adobe; plywood; cardboard; reused wood; wood planks or shingles; other	Cement; stone with lime or cement; bricks; cement blocks; covered adobe	Earth; sand; dung; wood planks; palm, bamboo; other	Parquet; polished wood; vinyl; asphalt strips; ceramic tiles; cement; carpet	(1) source of drinking water; (2) toilet facility; (3) cooking fuel; (4) electricity; ownership of a: (5) radio, (6) television, (7) refrigerator, (8) bicycle, (9) scooter, (10) mobile telephone, (11) watch, (12) bank account.
Burkina Faso 2010	DHS	No roof; thatch; palm leaf; sod; rustic mat; palm; bamboo; wood planks; wood; roofing shingles; other	Metal; zinc with cement fibre; tiles; slate; cement	No walls; cane; palm; trunk; dirt; bamboo with mud; stone with mud; uncovered adobe; wood planks or shingles; other	Cement; stone with lime or cement; bricks; cement blocks; covered adobe	Earth; sand; dung; wood planks; palm; bamboo; other	Parquet or polished wood; ceramic tiles; cement; carpet	(1) source of drinking water; (2) toilet facility; (3) cooking fuel; (4) electricity; ownership of a: (5) radio, (6) television, (7) bicycle, (8) scooter, (9) mobile telephone, (10) watch, (11) animal-drawn cart, (12) bank account.
Burkina Faso 2014	MIS	Thatch; palm leaf; wood; other	Metal	Cane; palm; trunks; dirt; bamboo with mud; stone with mud; uncovered adobe; wood planks or shingles; other	Cement; stone with lime or cement; bricks; cement blocks; covered adobe	Earth; sand; dung; other	Ceramic tiles; cement	(1) source of drinking water; (2) toilet facility; (3) cooking fuel; (4) electricity; ownership of a: (5) radio, (6) television, (7) bicycle, (8) scooter, (9) mobile telephone, (10) watch, (11) animal-drawn cart, (12) bank account
Burundi 2012	MIS	No roof; thatch; palm leaf; sod; palm; bamboo; wood; roofing shingles; other	Metal; ceramic tiles; cement	No walls; bamboo; palm; trunk; dirt; bamboo with mud; stone with mud; mud bricks; reused wood; wood planks or shingles	Cement; stone with lime or cement; bricks; cement blocks; covered adobe	Earth; sand; dung; other	Ceramic tiles; cement; carpet	(1) source of drinking water; (2) toilet facility; (3) cooking fuel; (4) electricity; ownership of a: (5) radio, (6) television, (7) bicycle, (8) mobile telephone, (9) watch, (10) bank account.
Cameroon 2011	DHS	No roof; thatch; palm leaf; sod; straw; rustic mat; palm; bamboo; wood planks; cardboard; wood; roofing shingles; other	Metal; calamine; cement fibre; ceramic tiles; cement	No walls; cane; palm; trunks; dirt; bamboo with mud; stone with mud; uncovered adobe; plywood; cardboard; reused wood; wood planks or shingles; other	Cement; stone with lime or cement; bricks; cement blocks; covered adobe	Earth; sand; dung; wood planks; palm; bamboo; other	Parquet; polished wood; vinyl; asphalt strips; ceramic tiles; cement; carpet	(1) source of drinking water; (2) toilet facility; (3) cooking fuel; (4) electricity; ownership of a: (5) radio, (6) television, (7) refrigerator, (8) bicycle, (9) scooter, (10) car, (11) mobile telephone, (12) watch, (13) bank account.

S2 Appendix. Classification of house type and development of the modified wealth index (continued)

Country	Survey type	Roof		Walls		Floor		Wealth index variables
		Rudimentary	Non-rudimentary	Rudimentary	Non-rudimentary	Rudimentary	Non-rudimentary	
Côte d'Ivoire 2011-12	DHS	No roof; thatch; palm leaf; sod; rustic mat; palm; bamboo; wood planks; wood; cardboard; roofing shingles; other	Metal; ceramic tiles; cement	No walls; cane; palm; trunk; dirt; bamboo with mud; stone with mud; uncovered adobe; plywood; cardboard; reused wood; wood planks or shingles; other	Cement; stone with lime or cement; bricks; cement blocks; covered adobe	Earth; sand; dung; wood planks; palm; bamboo; other	Parquet; polished wood; vinyl; asphalt strips; ceramic tiles; cement; carpet	(1) source of drinking water; (2) toilet facility; (3) cooking fuel; (4) electricity; ownership of a: (5) radio, (6) television, (7) refrigerator, (8) bicycle, (9) scooter, (10) mobile telephone, (11) watch, (12) bank account.
Democratic Republic of the Congo 2013-14	DHS	No roof; thatch; palm leaf; earth; rustic mat; palm; bamboo; wood planks; wood; shingles; other	Metal; zinc or cement fibre; tiles; slate; cement	No walls; cane; palm; trunks; dirt; bamboo with mud; stone with mud; uncovered adobe; bamboo; reused wood; wood; wood planks or shingles; other	Cement; stone with lime or cement; bricks; cement blocks; covered adobe	Earth; sand; dung; wood planks; palm; bamboo; other	Vinyl; asphalt strips; ceramic tiles; cement; carpet	(1) source of drinking water; (2) toilet facility; (3) cooking fuel; (4) electricity; ownership of a: (5) radio, (6) television, (7) bicycle, (8) mobile telephone, (9) watch
Gambia 2013	DHS	No roof; thatch; palm leaf; palm; bamboo; wood planks; cardboard; wood; roofing shingles; other	Metal; calamine or cement fibre; ceramic tiles; cement	No walls; cane; palm; trunks; mud; mud bricks; bamboo with mud; stone with mud; plywood; wood planks or shingles; other	Cement; stone with lime or cement; bricks; cement blocks	Earth; sand; wood planks; other	Parquet; polished wood; vinyl; asphalt strips; ceramic tiles; cement; carpet; plastic carpet	(1) source of drinking water; (2) toilet facility; (3) cooking fuel; (4) electricity; ownership of a: (5) radio, (6) television, (7) refrigerator, (8) bicycle, (9) scooter, (10) car, (11) mobile telephone, (12) watch, (13) animal-drawn cart
Ghana 2014	DHS	No roof; thatch; palm leaf; rustic mat; palm; bamboo; wood planks; cardboard; wood; roofing shingles; other	Metal; calamine; cement fibre; ceramic tiles; cement; asbestos; slate	No walls; cane; palm; trunks; dirt; bamboo with mud; stone with mud; uncovered adobe; plywood; cardboard; reused wood; wood planks or shingles; other	Cement; stone with lime or cement; bricks; cement blocks; covered adobe	Earth; sand; dung; wood planks; other	Parquet; polished wood; vinyl; asphalt strips; ceramic; marble; porcelain; cement; carpet; linoleum	(1) source of drinking water; (2) toilet facility; (3) cooking fuel; (4) electricity; ownership of a: (5) radio, (6) television, (7) refrigerator, (8) bicycle, (9) scooter, (10) car, (11) mobile telephone, (12) watch, (13) bank account.
Guinea 2012	DHS	No roof; thatch; palm leaf; rustic mat; wood; other	Metal; zinc or cement fibre; tiles; slate; cement	No walls; cane; palm; trunks; dirt; bamboo, wood or stone with mud; other	Cement; stone with lime or cement; cement blocks	Earth; sand; dung; palm; bamboo; other	Parquet; polished wood; vinyl; asphalt strips; ceramic tiles; cement	(1) source of drinking water; (2) toilet facility; (3) cooking fuel; (4) electricity; ownership of a: (5) radio, (6) television, (7) refrigerator, (8) bicycle, (9) scooter, (10) car, (11) mobile telephone, (12) bank account.

S2 Appendix. Classification of house type and development of the modified wealth index (continued)

Country	Survey type	Roof		Walls		Floor		Wealth index variables
		Rudimentary	Non-rudimentary	Rudimentary	Non-rudimentary	Rudimentary	Non-rudimentary	
Kenya 2015	MIS	No roof; thatch; grass; makuti; dung; mud; sod; other	Iron sheets; tin cans; asbestos sheet; concrete; tiles	No walls; cane; palm; trunks; dung; mud; sod; bamboo with mud; stone with mud; uncovered adobe; plywood; cardboard; reused wood; wood planks or shingles; other	Iron sheets; cement; stone with lime or cement; bricks; cement blocks; covered adobe	Earth; sand; dung; wood planks; other	Parquet or polished wood; vinyl; asphalt; ceramic tiles; cement; carpet	(1) source of drinking water; (2) toilet facility; (3) electricity; ownership of a: (4) radio, (5) television, (6) refrigerator; (7) bicycle, (8) scooter, (9) car, (10) mobile telephone, (11) watch, (12) bank account
Liberia 2009	MIS	Thatch; palm leaf; palm; bamboo; mats; tarpaulin; plastic; other	Zinc; metal; ceramic tiles; concrete; cement; asbestos	Mud and sticks; cane; palm; trunks; straw; thatch mats; mud bricks; cardboard; plastic; wood planks or shingles; other	Cement or stone blocks; bricks; zinc	Earth; sand; mud; wood planks; other	Parquet; polished wood; floor mat; linoleum; ceramic tiles; concrete; cement; carpet	(1) source of drinking water; (2) toilet facility; (3) cooking fuel; ownership of a: (4) radio, (5) television, (6) mobile telephone; (7) watch
Liberia 2011	MIS	Thatch; palm leaf; palm; bamboo; wood planks; tarpaulin; plastic; wood	Metal; ceramic tiles; concrete; cement; asbestos sheets	Mud and sticks; cane; palm; trunk; straw or thatched mats; mud bricks, plywood; cardboard; plastic; reused wood; wood planks or shingles; other	Cement; stone blocks; bricks; zinc	Earth; sand; mud; wood planks; other	Parquet; polished wood; floor mat; linoleum; ceramic tiles; concrete; cement; carpet	(1) source of drinking water; (2) toilet facility; (3) cooking fuel; ownership of a: (4) radio, (5) television, (6) scooter, (7) mobile telephone, (8) watch, (9) bank account.
Madagascar 2011	MIS	No roof; thatch; palm leaf; sod; rustic mat; palm with bamboo; wood planks; cardboard; wood; roofing shingles; other	Sheet metal; zinc or cement fibre; ceramic tiles; cement	No walls; cane; palm; trunks; dirt; bamboo with mud; stone with mud; plywood; cardboard; reused wood; wood planks or shingles; other	Cement; stone with lime or cement; bricks; cement blocks; sheet metal; tiles; ceramic	Mud; earth; dung; wood planks; palm; bamboo; mat; other	Parquet; polished wood; vinyl; asphalt strips; ceramic tiles; cement; carpet	(1) source of drinking water; (2) toilet facility; (3) cooking fuel; (4) electricity; ownership of a: (5) radio, (6) television, (7) bicycle, (8) mobile telephone, (9) watch, (10) animal-drawn cart (11) bank account.
Madagascar 2013	MIS	No roof; thatch; palm; leaf; sod; rustic mat; palm; bamboo; wood planks; cardboard; wood; roofing shingles; other	Sheet metal; zinc or cement fibre; ceramic tiles; cement	No walls; cane; palm; trunks; dirt; bamboo with mud; stone with mud; plywood; cardboard; reused wood; wood planks or shingles; other	Cement; stone with lime or cement; bricks; cement blocks	Mud; earth; dung; wood planks; palm; bamboo; mat; other	Parquet; polished wood; vinyl; asphalt strips; ceramic tiles; cement; carpet	(1) source of drinking water; (2) toilet facility; (3) cooking fuel; (4) electricity; ownership of a: (5) radio, (6) television, (7) bicycle, (8) mobile telephone, watch, (9) animal-drawn cart, (10) bank account.

S2 Appendix. Classification of house type and development of the modified wealth index (continued)

Country	Survey type	Roof		Walls		Floor		Wealth index variables
		Rudimentary	Non-rudimentary	Rudimentary	Non-rudimentary	Rudimentary	Non-rudimentary	
Malawi 2012	MIS	No roof; thatch; palm leaf; palm, bamboo; grass; wood planks; cardboard; wood; roofing shingles; other	Iron sheets; calamine or cement fibre; ceramic tiles; cement	No walls; cane; palm; trunks; dirt; bamboo or tree trunks with mud; stone with mud; plywood; cardboard; reused wood; other	Cement; stone with lime or cement; burnt bricks; unburnt bricks; cement blocks	Earth; sand; dung; broken bricks	Parquet; polished wood; vinyl; asphalt strips; ceramic tiles; cement; carpet	(1) source of drinking water; (2) toilet facility; (3) cooking fuel; (4) electricity; ownership of a: (5) radio, (6) television, (7) refrigerator, (8) bicycle, (9) mobile telephone, (10) bank account.
Malawi 2014	MIS	No roof; thatch; palm leaf; rustic mat; palm; bamboo; grass; wood planks; wood; roofing shingles; other	Iron sheets; calamine or cement fibre; ceramic tiles; cement	No walls; cane; palm; trunks; dirt; bamboo with mud; stone with mud; plywood; cardboard; wood planks or shingles; other	Cement; stone with lime or cement; burnt bricks; unburnt bricks; cement blocks	Earth; sand; dung; wood planks; broken bricks; other	Parquet; polished wood; vinyl; asphalt strips; ceramic tiles; cement; carpet	(1) source of drinking water; (2) toilet facility; (3) cooking fuel; (4) electricity; ownership of a: (5) radio, (6) television, (7) refrigerator, (8) bicycle, (9) mobile telephone, (10) bank account.
Mali 2012-13	DHS	No roof; thatch; palm leaf; sod; rustic mat; palm; bamboo; wood planks; cardboard; wood; roofing shingles; other	Metal; calamine or cement fibre; ceramic tiles; cement	No walls; cane; palm; trunk; dirt; bamboo with mud; stone with mud; uncovered adobe; plywood; cardboard; reused wood; wood planks or shingle; other	Cement; stone with lime or cement; bricks; cement blocks; covered adobe	Earth; sand; dung; wood planks; palm; bamboo; other	Parquet; polished wood; vinyl; asphalt strips; ceramic tiles; cement; carpet	(1) source of drinking water; (2) toilet facility; (3) cooking fuel; (4) electricity; ownership of a: (5) radio, (6) television, (7) refrigerator, (8) bicycle, (9) scooter, (10) mobile telephone, (11) watch, (12) animal-drawn cart, (13) bank account.
Mozambique 2011	DHS	No roof; grass; thatch; palm; other	Metal; calamine or cement fibre; ceramic tiles; concrete slab; cement	No walls; cane; palm; trunk; tin/cardboard/paper; sticks; shells (casca); wood or metal planks; adobe; other	Bricks; cement blocks	Earth; wood planks; adobe; other	Parquet; polished wood; tiles; bricks; cement	(1) source of drinking water; (2) toilet facility; (3) cooking fuel; (4) electricity; ownership of a: (5) radio, (6) television, (7) refrigerator, (8) bicycle, (9) scooter, (10) car, (11) mobile telephone, (12) watch, (13) bank account.
Nigeria 2010	MIS	Thatch; palm leaf; palm; bamboo; mats; wood planks; tarpaulin; plastic; wood	Zinc; metal; ceramic tiles; concrete; cement; asbestos	Mud and sticks; cane; palm; trunk; straw or thatched mats; mud bricks, plywood; reused wood; cardboard; plastic; wood planks or shingles; other	Cement or stone blocks; bricks	Earth; sand; wood planks	Parquet; polished wood; floor mat; linoleum; ceramic tiles; concrete; cement; carpet	(1) source of drinking water; (2) toilet facility; (3) cooking fuel; (4) electricity; ownership of a: (5) radio, (6) television, (7) refrigerator, (8) bicycle, (9) scooter, (10) car, (11) mobile telephone

S2 Appendix. Classification of house type and development of the modified wealth index (continued)

Country	Survey type	Roof		Walls		Floor		Wealth index variables
		Rudimentary	Non-rudimentary	Rudimentary	Non-rudimentary	Rudimentary	Non-rudimentary	
Rwanda 2010	DHS	No roof; thatch; palm leaf; sod; rustic mat; plastic; palm; bamboo; wood; roofing shingles; other	Metal; calamine or cement fibre; ceramic tiles; cement	No walls; cane; palm; trunk; dirt; bamboo with mud; stone with mud; uncovered adobe; plywood; reused wood; trunks with mud; wood planks or shingles; other	Cement; stone with lime or cement; bricks; cement blocks; covered adobe	Earth; sand; dung; wood planks; other	Ceramic tiles; cement	(1) source of drinking water; (2) toilet facility; (3) cooking fuel; (4) electricity; ownership of a: (5) radio, (6) television, (7) bicycle, (8) mobile telephone, (9) watch, (10) bank account.
Senegal 2008-09	MIS	No roof; thatch; palm leaf; sod; rustic mat; palm; bamboo; wood planks; cardboard; wood; roofing shingles; other	Metal; calamine; cement fibre; ceramic tiles; cement	No walls; cane; palm; trunks; dirt; bamboo with mud; stone with mud; uncovered adobe; plywood; reused wood; wood planks or shingles; other	Cement; stone with lime or cement; bricks; cement blocks; covered adobe	Earth; sand; dung; wood planks; palm; bamboo; other	Parquet; polished wood; vinyl; asphalt strips; ceramic tiles; cement; carpet	(1) source of drinking water; (2) toilet facility; (3) cooking fuel; (4) electricity; ownership of a: (5) radio, (6) television, (7) refrigerator, (8) bicycle, (9) scooter, (10) landline telephone, (11) mobile telephone, (12) animal-drawn cart
Senegal 2010-11	DHS	No roof; thatch; palm leaf; sod; rustic mat; palm; bamboo; wood planks; cardboard; wood; roofing shingles; other	Metal; calamine or cement fibre; ceramic tiles; cement	No walls; cane; palm; trunks; dirt; bamboo with mud; stone with mud; uncovered adobe; plywood; cardboard; reused wood; wood planks or shingles; other	Cement; stone with lime or cement; bricks; cement blocks; covered adobe	Earth; sand; dung; wood planks; palm; bamboo; other	Parquet; polished wood; vinyl; asphalt strips; ceramic tiles; cement; carpet	(1) source of drinking water; (2) toilet facility; (3) cooking fuel; (4) electricity; ownership of a: (5) radio, (6) television, (7) refrigerator, (8) bicycle, (9) scooter, (10) landline telephone; (11) mobile telephone, (12) animal-drawn cart; (13) bank account.
Senegal 2012-13	DHS	No roof; thatch; palm leaf; sod; palm; bamboo; wood planks; cardboard; wood; roofing shingles; other	Metal; calamine or cement fibre; ceramic tiles; cement	No walls; cane; palm; trunks; dirt; bamboo with mud; stone with mud; reused wood; wood planks or shingles; other	Cement; stone with lime or cement; bricks; cement blocks; covered adobe	Earth; sand; dung; wood planks; palm; bamboo; other	Parquet; polished wood; vinyl; asphalt strips; ceramic tiles; cement; carpet	(1) source of drinking water; (2) toilet facility; (3) cooking fuel; (4) electricity; ownership of a: (5) radio, (6) television, (7) refrigerator, (8) bicycle, (9) scooter, (10) landline telephone, (11) mobile telephone, (12) animal-drawn cart, (13) bank account.
Senegal 2013-14	DHS	No roof; thatch or palm leaf; sod; rustic mat; palm or bamboo; wood planks; wood; roofing shingles; other	Metal; calamine or cement fibre; ceramic tiles; cement	No walls; cane; palm; trunk; dirt; bamboo with mud; stone with mud; reused wood; wood planks or shingles; other	Cement; stone with lime or cement; bricks; cement blocks; covered adobe	Earth; sand; dung; wood planks; palm; bamboo; other	Parquet; polished wood; vinyl; asphalt strips; ceramic tiles; cement; carpet	(1) source of drinking water; (2) toilet facility; (3) cooking fuel; (4) electricity; ownership of a: (5) radio, (6) television, (7) refrigerator, (8) bicycle, (9) scooter, (10) car; (11) mobile telephone, (12) animal-drawn cart, (13) bank account.

S2 Appendix. Classification of house type and development of the modified wealth index (continued)

Country	Survey type	Roof		Walls		Floor		Wealth index variables
		Rudimentary	Non-rudimentary	Rudimentary	Non-rudimentary	Rudimentary	Non-rudimentary	
Togo 2013-14	DHS	No roof; thatch; palm leaf; sod; palm; bamboo; wood planks; cardboard; straw; wood; roofing shingles; other	Metal; calamine or cement fibre; ceramic tiles; cement	No walls; cane; palm; trunk; dirt; bamboo with mud; stone with mud; uncovered adobe; plywood; cardboard; reused wood; wood planks or shingles; other	Cement; stone with lime or cement; bricks; cement blocks; covered adobe	Earth; sand; dung; palm; bamboo; other	Parquet; polished wood; vinyl; asphalt strips; ceramic tiles; cement; carpet	(1) source of drinking water; (2) toilet facility; (3) cooking fuel; (4) electricity; ownership of a: (5) radio, (6) television, (7) refrigerator, (8) bicycle, (9) scooter, (10) mobile telephone, (11) watch, (12) bank account.
Uganda 2009	MIS	Thatched; mud; wood; planks; other	Iron sheets; asbestos; tiles; tin; cement	Thatched; straw; mud and poles; unburnt bricks; unburnt bricks with plaster; burnt bricks with mud; timber; other	Cement blocks; stone; burnt bricks with cement	Earth; sand; dung; other	Mosaic; tiles; bricks; cement; stones	(1) source of drinking water; (2) toilet facility; (3) cooking fuel; (4) electricity; ownership of a: (5) radio, (6) television, (7) bicycle, (8) scooter, (9) mobile telephone, (10) watch; (11) bank account
Uganda 2014-15	MIS	Thatched; mud; palm; wood planks; wood; roofing shingles; other	Tin; iron sheets; cement fibre; tiles; cement; asbestos	No walls; thatched; straw; dirt; mud and poles; stone with mud; reused wood; unburnt bricks (including with plaster or mud); wood planks or shingles; other	Cement; stone with lime or cement; burnt bricks with cement; cement blocks	Earth; sand; dung; wood planks; other	Parquet; polished wood; mosaic; tile; cement; stones; bricks	(1) source of drinking water; (2) toilet facility; (3) cooking fuel; (4) electricity; ownership of a: (5) radio, (6) television, (7) refrigerator; (8) bicycle, (9) scooter, (10) mobile telephone, (11) watch, (12) bank account

Improved sanitation facilities: flush toilet, piped sewer system, septic tank, flush/pour flush to pit latrine, ventilated improved pit latrine, pit latrine with slab or composting toilet [1]. Improved sources of drinking water: piped water into dwelling, piped water to yard/plot, a public tap or standpipe, tubewell or borehole, protected dug well, protected spring or rainwater [1]. Modern energy sources: liquefied petroleum gas, biogas, ethanol gel, plant oils, dimethyl ether, electricity; intermediate energy sources: charcoal, coal, kerosene; traditional energy sources: dung, agricultural residues, straw and wood [2].

References

1. JMP. Definitions and methods: Improved and unimproved water sources and sanitation facilities. Geneva: WHO/UNICEF Joint Monitoring Programme (JMP) for Water Supply and Sanitation; 2016.
2. IEA. Energy for cooking in developing countries. Paris: International Energy Agency; 2006.
