# Supplementary material for: Housing Improvements and Malaria Risk in Sub-Saharan Africa: A Multi-Country Analysis of Survey Data
Source: PLoS Med. 2017 Feb 21;14(2):e1002234. doi: 10.1371/journal.pmed.1002234 (PMC5319641; doi:10.1371/journal.pmed.1002234)
Supplement: S3 Appendix — (PDF) [file pmed.1002234.s003.pdf]

S3 Appendix. Association between house type, ITN use and malaria infection in children aged 0-5 years in sub-Saharan Africa (unadjusted analysis).

Table S3. Malaria infection prevalence in children aged 0-5 years stratified by house type and ITN use

Country	Survey type	PR by microscopy (Total blood smears)				PR by RDT (Total RDTs)			
		House type		Slept under ITN the previous night		House type		Slept under ITN the previous night	
		Modern	Traditional	Yes	No	Modern	Traditional	Yes	No
Angola 2011	MIS	2.4 (995)	12.8 (2436)	5.1 (818)	11.2 (2613)	2.5 (996)	16.7 (2436)	7.7 (818)	14.1 (2614)
Benin 2011-12	DHS	22.5 (1695)	35.0 (2943)	30.2 (3449)	31.3 (1189)	17.8 (1711)	33.2 (2984)	26.7 (3490)	30.0 (1205)
Burkina Faso 2010	DHS	45.5 (1371)	70.6 (4731)	62.2 (3095)	67.8 (3007)	61.2 (1376)	79.8 (4749)	74.5 (3108)	76.8 (3017)
Burkina Faso 2014	MIS	31.0 (1079)	51.2 (5038)	46.9 (4672)	50.1 (1445)	46.9 (1094)	68.2 (5060)	64.0 (4702)	66.0 (1452)
Burundi 2012	MIS	2.0 (458)	18.1 (3264)	12.1 (2039)	21.1 (1683)	2.0 (458)	23.2 (3292)	15.5 (2042)	26.7 (1708)
Cameroon 2011	DHS	-	-	-	-	27.4 (2437)	37.1 (4168)	28.9 (1656)	35.0 (4949)
Côte d'Ivoire 2011-12	DHS	13.3 (2331)	23.7 (1713)	17.1 (1576)	18.1 (2468)	37.2 (2415)	59.1 (1800)	44.2 (1623)	48.0 (2592)
DRC 2013-14	DHS	13.2 (838)	27.8 (7348)	23.0 (4392)	30.3 (3794)	17.9 (859)	38.1 (7360)	32.7 (4407)	39.9 (3812)
Gambia 2013	DHS	0.5 (1841)	0.4 (1640)	0.4 (1697)	0.6 (1784)	2.0 (1736)	1.5 (1562)	1.2 (1620)	2.4 (1678)
Ghana 2014	DHS	26.1 (1931)	37.8 (1266)	33.9 (1505)	27.9 (1692)	34.3 (1927)	54.3 (1264)	47.0 (1502)	37.9 (1689)
Guinea 2012	DHS	30.7 (1362)	52.7 (1858)	44.9 (933)	42.8 (2287)	28.9 (1360)	57.8 (1855)	47.6 (931)	44.7 (2284)
Kenya 2015	MIS	3.0 (1280)	7.2 (2825)	6.3 (2298)	5.4 (1807)	4.7 (1278)	12.5 (2817)	11.9 (2294)	7.7 (1801)
Liberia 2009	MIS	23.0 (1195)	36.2 (3773)	31.3 (1429)	33.7 (3539)	27.4 (1193)	40.1 (3767)	33.5 (1428)	38.5 (3532)
Liberia 2011	MIS	18.3 (798)	36.7 (2283)	30.6 (1143)	32.8 (1938)	34.5 (812)	57.1 (2375)	50.7 (1190)	51.7 (1997)
Madagascar 2011	MIS	0.4 (804)	4.8 (6032)	4.9 (5172)	2.5 (1664)	1.6 (810)	7.0 (6064)	6.8 (5201)	5.1 (1673)
Madagascar 2013	MIS	0.9 (462)	7.8 (5689)	8.5 (3323)	5.8 (2828)	2.1 (468)	8.7 (5764)	9.4 (3368)	6.8 (2864)
Malawi 2012	MIS	9.3 (526)	29.9 (1586)	21.9 (1240)	28.9 (872)	15.0 (526)	46.2 (1589)	36.2 (1241)	41.6 (874)
Malawi 2014	MIS	12.8 (562)	32.3 (1366)	25.0 (1351)	30.3 (577)	13.0 (560)	37.1 (1361)	28.0 (1345)	34.9 (576)
Mali 2012-13	DHS	16.2 (884)	56.5 (4762)	49.9 (3954)	50.8 (1692)	11.2 (902)	51.3 (4804)	45.0 (3991)	44.9 (1715)
Mozambique 2011	DHS	7.1 (1019)	35.9 (3879)	31.0 (1629)	29.4 (3269)	7.4 (1026)	40.9 (3890)	34.2 (1636)	33.8 (3280)
Nigeria 2010	MIS	30.1 (2117)	43.7 (3020)	36.5 (1549)	38.8 (3588)	38.1 (2117)	53.5 (3030)	47.7 (1558)	46.9 (3589)
Rwanda 2010	DHS	0.6 (642)	1.6 (4308)	1.2 (3338)	1.9 (1612)	1.1 (638)	2.8 (4255)	2.1 (3302)	3.6 (1591)
Senegal 2008-09	MIS	1.8 (1370)	9.2 (2768)	6.2 (1467)	7.0 (2671)	8.1 (1358)	13.9 (2674)	11.2 (1425)	12.4 (2607)
Senegal 2010-11	DHS	2.8 (1947)	4.9 (2751)	3.8 (2036)	4.1 (2662)	2.4 (1957)	4.2 (2759)	3.4 (2043)	3.5 (2673)
Senegal 2012-13	DHS	1.4 (3222)	6.4 (4044)	3.4 (3615)	4.9 (3651)	2.0 (3229)	6.8 (4087)	3.7 (3638)	5.7 (3678)
Senegal 2013-14	DHS	0.5 (2944)	2.9 (3818)	1.6 (3439)	2.2 (3323)	0.3 (2944)	3.0 (3818)	1.6 (3440)	2.0 (3322)
Togo 2013-14	DHS	25.2 (1597)	49.5 (2291)	37.3 (1690)	41.3 (2198)	27.0 (1586)	51.0 (2282)	38.7 (1677)	43.0 (2191)
Uganda 2009	MIS	23.9 (716)	47.8 (3295)	36.6 (1356)	47.1 (2655)	32.0 (712)	57.6 (3286)	47.8 (1351)	55.7 (2647)
Uganda 2014-15	MIS	7.7 (970)	22.7 (3969)	20.4 (3760)	17.5 (1179)	13.9 (964)	37.4 (3939)	34.0 (3736)	28.8 (1167)

DHS: Demographic and Health Survey; ITN: insecticide-treated net; MIS: Malaria Indicator Survey; PR: parasite rate; RDT: rapid diagnostic test.

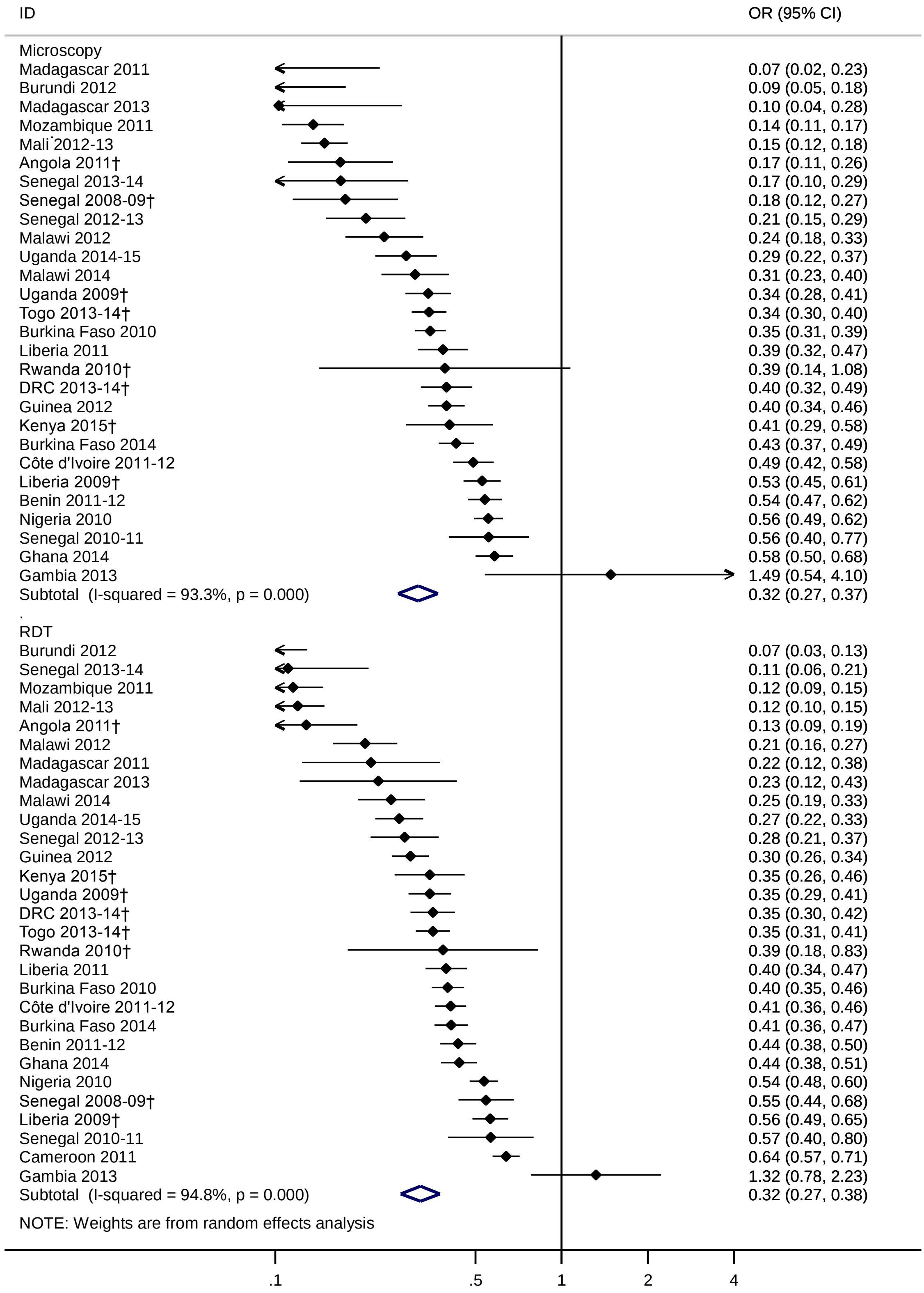

Figure S3a. Association between house type and malaria infection in children aged 0-5 years in sub-Saharan Africa (unadjusted analysis). The pooled reduction in odds of malaria infection in modern housing, compared to traditional housing, is shown to the left of the vertical line representing the null value. Data are taken from 15 Demographic and Health Surveys and 14 Malaria Indicator Surveys conducted between 2008 and 2015. Houses built with a finished wall, finished roof and finished floor material were classified as modern and all other houses were classified as traditional (S2 Appendix). Summary effects are from random-effects analysis. Sub-groups show diagnostic test. Error bars show 95% confidence intervals.

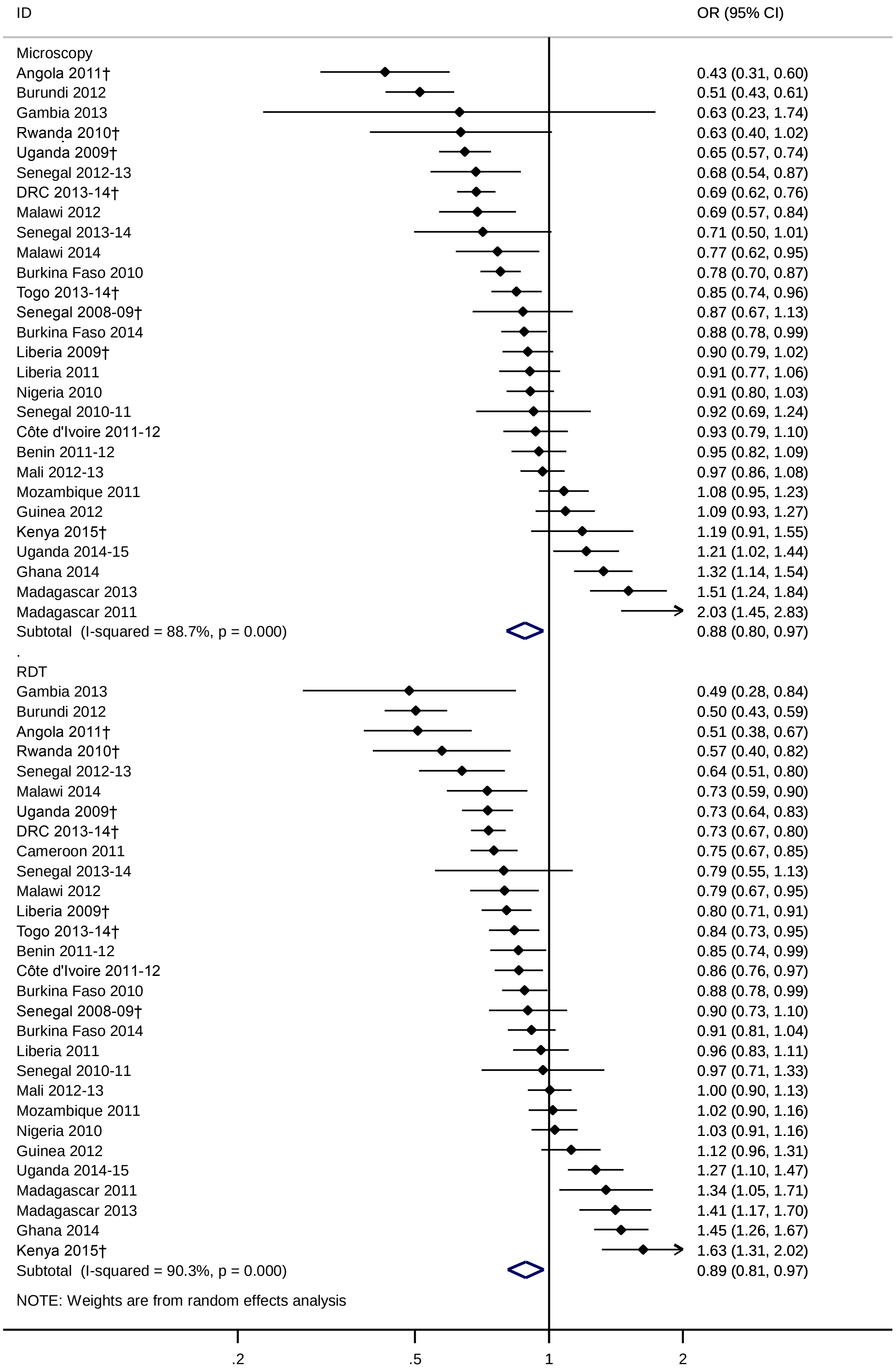

Figure S3b. Association between insecticide-treated net (ITN) use and malaria infection among children aged 0-5 years in sub-Saharan Africa (unadjusted analysis). The pooled reduction in odds of malaria infection in ITN users, compared to non-users, is shown to the left of the vertical line representing the null value. Data are taken from 15 Demographic and Health Surveys and 14 Malaria Indicator Surveys conducted between 2008 and 2015. Summary effects are from random-effects analysis. Sub-groups show diagnostic test. Error bars show 95% confidence intervals.
