# Supplementary material for: Housing Improvements and Malaria Risk in Sub-Saharan Africa: A Multi-Country Analysis of Survey Data
Source: PLoS Med. 2017 Feb 21;14(2):e1002234. doi: 10.1371/journal.pmed.1002234 (PMC5319641; doi:10.1371/journal.pmed.1002234)
Supplement: S4 Appendix — (PDF) [file pmed.1002234.s004.pdf]

S4 Appendix. Association between house type and malaria infection in children aged 0-5 years in sub-Saharan Africa, stratified by ITN use.

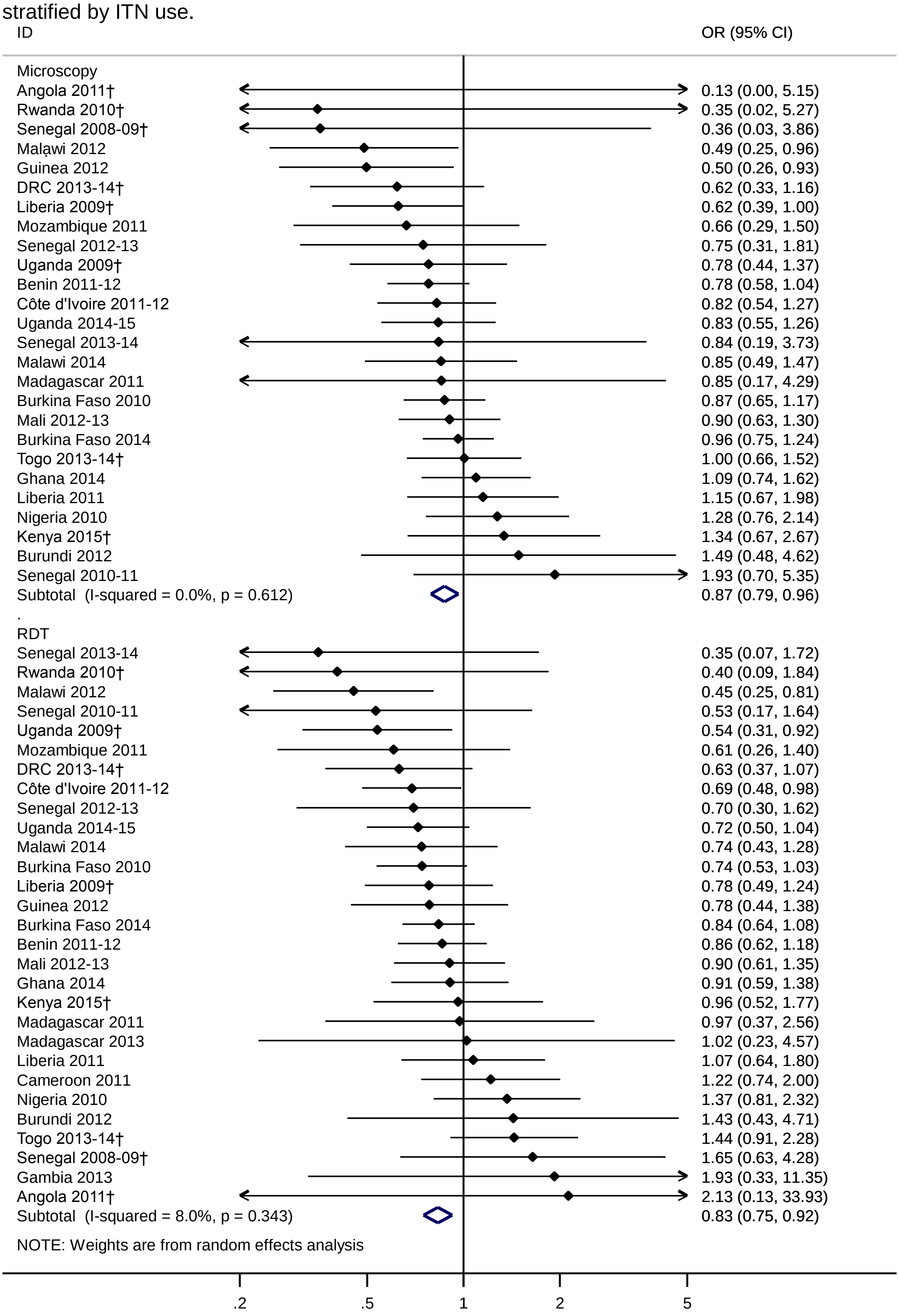

Figure S4a. Association between house type and malaria infection among children aged 0-5 years sleeping under an insecticide treated net. The pooled reduction in odds of malaria infection in modern housing, compared to traditional housing, is shown to the left of the vertical line representing the null value. Odds ratios (OR) are adjusted for age, gender, indoor residual spraying (IRS; where measured), household wealth and geographic cluster. Summary effects are from random effects analysis. Sub-groups show diagnostic test. Error bars show 95% confidence intervals. †OR not adjusted for IRS.

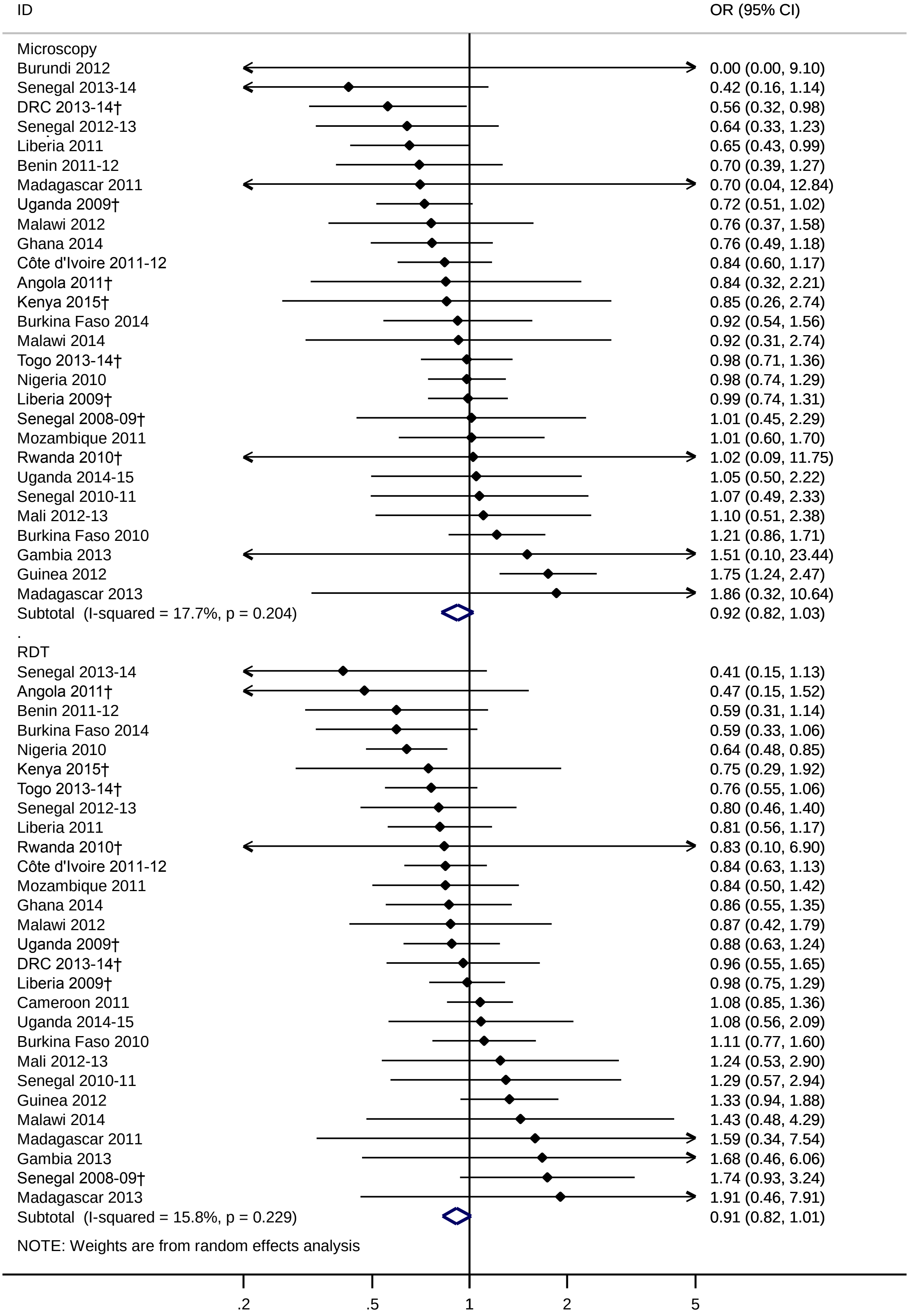

Figure S4b. Association between house type and malaria infection among children aged 0-5 years not sleeping under an insecticide treated net. The pooled reduction in odds of malaria infection in modern housing, compared to traditional housing, is shown to the left of the vertical line representing the null value. Odds ratios (OR) are adjusted for age, gender, indoor residual spraying (IRS; where measured), household wealth and geographic cluster. Summary effects are from random effects analysis. Sub-groups show diagnostic test. Error bars show 95% confidence intervals. †OR not adjusted for IRS.
